# Parent Outcomes from a Randomized Controlled Trial Investigating a Modular Behavioral Intervention for Young Autistic Children

**DOI:** 10.1002/aur.70013

**Published:** 2025-02-24

**Authors:** Lynne Levato, Samantha Hochheimer, Hongyue Wang, Lisa Wallace, Susan Hyman, Cynthia Anderson, Zachary Warren, Eric Butter, Ryan Martin, Evon Lee, Tristram Smith, Cynthia Johnson

**Affiliations:** ^1^ University of Rochester Medical Center Rochester New York USA; ^2^ Vanderbilt University Medical Center Nashville Tennessee USA; ^3^ The May Institute Randolph Massachusetts USA; ^4^ Nationwide Children's Hospital Columbus Ohio USA; ^5^ Case Western Reserve University and Cleveland Clinic Cleveland Ohio USA

**Keywords:** applied behavior analysis, autism, behavioral intervention, parent training, parental competence, parental stress, randomized clinical trial (RCT)

## Abstract

We assessed parent stress and competence outcomes from participation in a randomized controlled trial of a modular behavioral intervention (Modular Approach for Young Autistic Children; MAYAC) compared to a treatment‐as‐usual comprehensive behavioral intervention (CBI). Throughout their participation, parents of military families were included in their child's treatment (e.g., identifying goals, learning strategies to support their child) and reported on their feelings of stress using the Parenting Stress Index—4, Short Form (PSI‐4), as well as their feelings of satisfaction and efficacy as a parent on the Parenting Sense of Competence (PSOC) scale. A linear mixed model evaluated the differences in stress and competence from baseline to each assessment period through follow‐up. There were no significant differences between groups in stress or competence ratings; however, there were within‐group changes in both treatment arms over the course of the trial. In both groups, parent stress decreased, and competence increased over time, continuing to suggest that behavioral analytic intervention for young children with autism can promote positive parent outcomes.

**Trial Registration:** ClinicalTrial.gov identifier: NCT04078061

**Summary**

- We asked parents of autistic children from military families to report on their parenting stress and feelings of competence and effectiveness as a parent throughout a 24‐week behavioral intervention program and a 6‐month follow‐up period.
- Young children with autism were randomly assigned to one of two programs: (1) a lower dose program with modifications made based on the child's progress or (2) a more traditional, comprehensive behavioral intervention of more hours.
- Both programs allowed for parents to be involved in helping make decisions about their child's goals, and parents also were able to learn strategies to help their child.
- Parents in both programs reported lower stress and greater feelings of parenting effectiveness throughout their participation in this study.
- These findings suggest that involving parents in their autistic child's behavioral intervention program can promote positive parent outcomes.

## 1 Introduction

Military families raising autistic children can experience a compounding of stress and burdens. More generally, parents of autistic children often report higher levels of stress than parents of neurotypical children (DesChamps et al. 2020; Iadarola et al. 2018; Rivard et al. 2014). This has been well documented worldwide and among diverse autistic populations (Al‐Farsi et al. 2016; Fairthorne et al. 2015; Hickey et al. 2021; Pisula and Porębowicz‐Dorsmann 2017), including military families (Davis and Finke 2015). Unique stressors for military families can include frequent relocations, isolation from family and other social supports, partner separation due to deployment, and financial constraints (Christi et al. 2023; Huebner 2019; Skomorovsky et al. 2016). For those families with autistic children, additional stress may be incurred when there is a lack of autism providers and long waitlists for intervention services, such as comprehensive behavioral interventions (CBI; Christi et al. 2023).

CBI is frequently sought to increase skills in communication, social interaction, and adaptive behavior in early childhood. Although traditionally clinician‐led, parent involvement is a component of CBI models. Considerable empirical support for parent training on focused areas, whether that be social communication targets, sleep, or disruptive behaviors, has emerged over the past 15 years (Iadarola et al. 2018; Johnson et al. 2019, 2023). There is increasing evidence that teaching parents of autistic children strategies to support their child's learning and development can lead to reductions in parental stress and an increased sense of competence, or feelings of satisfaction and efficacy as a parent (e.g., D'Entremont et al. 2022; Iadarola et al. 2018; Li et al. 2024). The extent of parent involvement varies across programs and ranges from information sharing with parents (Bearss et al. 2015) to exclusively parent‐mediated intervention (Ingersoll et al. 2016). Parent involvement also depends on the intervention model and can be impacted by provider availability, insurance coverage, geographic disparities, distance to clinic, and work schedules (Yingling et al. 2017). Other barriers include difficulty engaging families and a perception that families are not interested in participating in parent training (Straiton et al. 2021).

In practice, structured, manualized, parent training as a model is not widely utilized. In an Autism Care Demonstration Annual Report from 2024, military family beneficiaries who received applied behavior analysis (ABA) services engaged in an average of 8.1 family treatment hours annually, with 40% of families receiving at least one session per month (Department of Defense 2024). In a national survey of 1089 behavior analysts, only 15% of ABA providers reported that they used a manualized parent training program with their clients (Ingersoll et al. 2020). Ingersoll and colleagues speculated that this low rate might be due in part to the fact that ABA providers are trained to provide highly individualized interventions, and they are less trained and therefore less comfortable in providing coaching as a part of parent‐mediated interventions (2020). Thus, manualized models that incorporate parents in intervention decision‐making and accompanying parent training may be appealing to many providers.

In a recent randomized control trial (RCT) comparing two behavioral intervention approaches for autistic children of military families (Anderson et al. 2024), we compared outcomes between a more intensive direct behavioral intervention (CBI) and an intervention with increased parent involvement and fewer intervention hours (MAYAC) and found that MAYAC was no less effective in improving adaptive behaviors over the 6 months of the trial. The purpose of this study is to assess parent outcomes from the RCT. Because MAYAC was designed to be delivered across fewer hours and with more structured parental involvement, we hypothesized that at the end of the 24‐week intervention and at the 48‐week follow‐up, MAYAC would be superior to CBI in decreasing parent stress and increasing parent sense of competence.

## 2 Methods

### 2.1 Design and Participating Sites

This single‐blind RCT was approved and overseen by the University of Rochester's' Institutional Review Board (IRB, central IRB of record) and the DOD Office of Human Research Protection, and registered with ClinicalTrial.gov as well as with the publicly accessible National Database Archive (NDA). Here, we analyzed parent outcome data from this trial comparing the effectiveness of MAYAC and a treatment‐asas‐usual CBI approach (see details in Anderson et al. 2024). There were four recruitment sites (Cleveland Clinic, May Institute, Nationwide Children's Hospital, and Vanderbilt University Medical Center) and a fifth site managed the administrative and data coordinating responsibilities (University of Rochester Medical Center). Participants who met inclusion criteria were invited to provide informed consent and then complete baseline assessments prior to randomization to MAYAC or CBI. Detailed information about trial design is available in Anderson et al. (2024).

### 2.2 Participants

Fifty‐six parents and their children with a diagnosis of autism between the ages of 18–59 months participated in the 24‐week intervention study delivered in the home or clinic settings and were assessed for follow‐up at week 48. In addition to the diagnosis and ages listed above, eligibility also required children to have approval or be in the process of being approved through TRICARE military insurance for ABA services (see Anderson et al. 2024 for more details). Our sample was unique as we focused exclusively on families who had at least one parent on active duty in the military.

### 2.3 Treatment Groups

#### 2.3.1 Modular Approach for Young Autistic Children (MAYAC)

All children randomized to MAYAC received up to 5 h of an intervention module called Social Communication and Engagement Intervention (SCEI) weekly throughout the trial. The first 4 weeks of the trial were exclusively SCEI, in which a child received 4.5 h per week of clinician‐led intervention focused on goals related to engagement, social communication, and interaction. In addition to the 4.5 h, the parent‐mediated component of SCEI was delivered for 30 min weekly, with sessions alternating between didactic instruction with role‐playing and active coaching of parents when they interacted with their children.

After the initial 4 weeks of intervention and then every 4 weeks thereafter, the dose and/or focus of intervention could be “tailored” to meet the individual needs of participants and their parents. Intervention tailoring involved several options, including (a) increasing the dose of SCEI, (b) adding additional goals to SCEI such as learning readiness or using augmentative communication, and (c) adding parent‐mediated intervention goals such as addressing sleep concerns, restrictive eating, toilet training, or challenging behavior (see Anderson et al. 2024 for details). MAYAC always consisted of a total of 5 h of SCEI, and the maximum dose was 10 h, with no more than 1.5 h of parent‐mediated intervention per week.

#### 2.3.2 Comprehensive Behavioral Intervention (CBI)

Participants in CBI were to receive 15–20 h of intervention, in line with traditional CBI. Intervention sessions included discrete trial training delivered for at least half of each session, and intervention goals were developed based on the Verbal Behavior Milestones Assessment and Placement Program (VB‐MAPP; Sundberg 2014). Goals included increasing communication, addressing social skills, and decreasing behavioral challenges (see Anderson et al. 2024 for more details).

Parent‐mediated intervention was also delivered to the CBI group in the manner commonly used at each site. As this was intended to be treatment as usual, we did not attempt to standardize the amount, content, or structure of parent training provided. Providers were instructed to deliver the CBI and any accompanying parent training as they would if the child were not enrolled in the study.

### 2.4 Parent Outcome Measures

The Parenting Stress Index—4, Short Form (PSI‐4 SF; Abidin 1995) is a 36‐item parent questionnaire rated on a five‐point scale (“Strongly Disagree” = 1 to “Strongly Agree” = 5). The PSI‐4 SF is a commonly used measure to assess parenting stress in this population (Iadarola et al. 2018; Johnson et al. 2019) and has good test–retest reliability (ICC = 0.77) and internal consistency (IC; 0.91; Barroso et al. 2016). The PSI‐4 SF yields four subscales and a total score (range 36–180), with higher scores reflecting higher stress. The Difficult Child (DC) subscale (range 12–60), which assesses stress related specifically to characteristics of the child, was chosen as our subscale of interest for this analysis of family outcomes. In addition, an exploratory analysis of the total score was also conducted.

The Parenting Sense of Competence (PSOC; Gibaud‐Wallston and Wandersman 1978) is a 17‐item parent questionnaire that assesses feelings of satisfaction and efficacy as a parent. Parents rate from “Strongly disagree” = 1 to “Strongly agree” = 6, and higher score totals (range 17–102) reflect more competence. This measure has high internal consistency and solid test–retest reliability and is sensitive to change in previous parent training studies (e.g., Iadarola et al. 2018; Johnson et al. 2023).

### 2.5 Statistical Analysis

Data were analyzed using the SAS 9.4 System for Windows. In accordance with the intent‐to‐treat principle, all randomized subjects were analyzed within the group to which they were assigned. We compared demographics as well as baseline patient and family characteristics between the study groups. Categorical variables were compared using the Chi‐Square or Fisher's exact test, and continuous variables were compared using the *t*‐test or Wilcoxon Rank Sum Test.

Since we hypothesized that through 48 weeks of follow‐up, MAYAC would be superior to CBI in decreasing parent stress, a linear mixed model (LMM) was fitted to analyze the parent stress outcomes (PSI‐4 SF Difficult Child Scale and Total Score, and PSOC) assessed at baseline through week 48. The LMM included fixed effects for the intercept, study group, time, and group‐by‐time interaction. The model also included random effects for the intercept and time, and an unstructured within‐person correlation structure for the residual errors. Changes from baseline to follow‐up were estimated and compared between the two groups to reflect the estimated treatment effect. The standard error of the treatment effect was estimated using a sandwich‐type robust variance estimator. All tests were two‐sided, and a *p*‐value less than 5% was considered significant.

## 3 Results

### 3.1 Demographics

In both groups, most primary informants identified as female (96.43%) with either some college or a college degree (75.00%), and partners serving in the military (89.29%). Children receiving intervention in both groups were primarily white (60.71%) and male (76.79%), with a mean age of about 34 months (SD = 10.27; see Table 1). There were no significant differences between the study groups.

**TABLE 1:** Demographics.

	No. (%)	
	MAYAC (*n* = 27)	CBI (*n* = 29)
Parent demographics		
Gender		
Female	25 (92.6)	29 (100.0)
Male	2 (7.4)	0 (0.0)
Mother age (years; mean, SD)	30.6 (6.2)	30.0 (6.0)
Father age (years; mean, SD)	31.7 (7.1)	31.8 (5.9)
Two‐parent family	27 (100.0)	26 (89.7)
Education		
High school grad	6 (22.2)	3 (10.3)
Some college	9 (33.3)	14 (48.3)
College graduate	10 (37.0)	9 (31.0)
Advanced degree	2 (7.4)	3 (10.3)
Military rank		
Enlisted (E1‐E9)	3 (11.1)	2 (6.9)
Officer (O1‐O10)	0 (0.0)	1 (3.4)
Not in military	24 (88.9)	26 (89.7)
Household income		
Less than $20,000	1 (3.7)	0 (0.0)
$20,001–$40,000	5 (18.5)	7 (24.1)
$40,001–$60,000	9 (33.3)	12 (41.4)
$60,001–$80,000	7 (25.9)	3 (10.3)
$80,001–$100,000	3 (11.1)	4 (13.8)
More than $100,000	2 (7.4)	3 (10.3)
Child demographics		
Gender		
Female	7 (25.9)	6 (20.7)
Male	20 (74.1)	23 (79.3)
Age (months; mean, SD)	33.2 (8.8)	34.7 (11.8)
Race		
African American/Black	7 (25.9)	2 (6.9)
White/Caucasian	14 (51.9)	20 (69.0)
Filipino/a	0 (0.0)	2 (6.9)
Multiracial	4 (14.8)	4 (13.8)
Other	2 (7.4)	1 (3.5)
Hispanic/Latinx	6 (22.2)	7 (24.1)

*Note*: Parent demographics are based on the primary informant.

### 3.2 Intervention Hours

**FIGURE 1:**
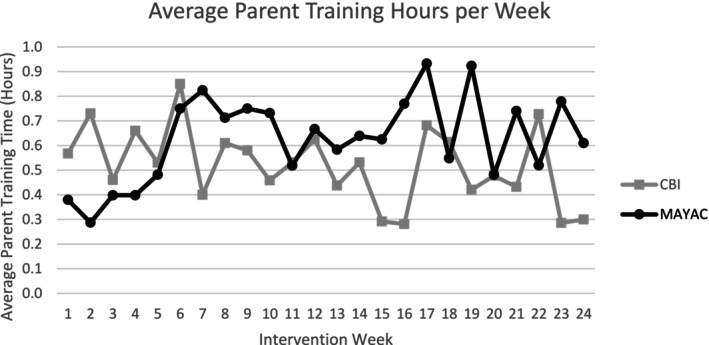
Average parent training hours by group.

During the 24‐week intervention, children in the CBI group received an average of 13.63 total hours of intervention per week (range 5.83–19.57 h), with approximately 31 min of that time being for parent‐mediated intervention (*M* = 0.52, SD = 0.68, range 0.00–6.75 h). Children in the MAYAC group received a weekly total average of 5.09 h of intervention (range 1.44–9.26 h), with approximately 38 min of that time for parent‐mediated intervention (*M* = 0.63, SD = 0.71, range 0.0–4.0 h). See Figure 1 for the average parent training hours for each group throughout the intervention. Results from a LMM showed no significant difference in the amount of parent training between the CBI and MAYAC groups (*p* = 0.1059).

### 3.3 PSI‐4 SF—Difficult Child (DC) Scale

**FIGURE 2:**
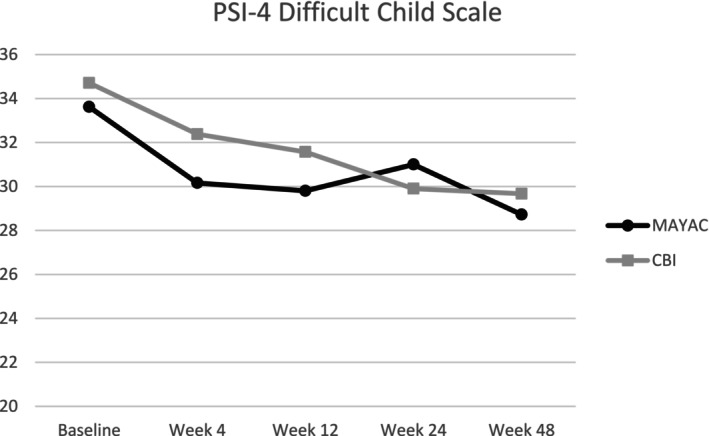
Changes in mean PSI‐4 difficult child scale scores.

Raw scores on the DC scale of the PSI‐4 were highest for both MAYAC and CBI groups at Baseline, indicating higher levels of stress prior to starting treatment. Over time, both groups saw a decrease in average stress (see Table 2). There were no significant group differences in parent stress on the DC scale between MAYAC and CBI at any time point, and the interaction effect between time and treatment was also not significant. The MAYAC group showed significant within‐group differences from Baseline to Week 4 (*p* = 0.0022), indicating that parents in MAYAC reported a decrease in stress during this time. This was not the case for the CBI group between Baseline and Week 4 (*p =* 0.1048). Within‐group differences showed lower stress from Baseline to Week 12, Week 24, and Week 48 in both MAYAC and CBI. Figure 2 provides a depiction of the trends of the PSI‐4 DC scores throughout the intervention and follow‐up, and Table 3 shows within‐group differences.

**TABLE 2:** Mean (SD) parenting stress and competence by group and timepoint.

	MAYAC (*n* = 27)					CBI (*n* = 29)				
	BL	Wk 4	Wk 12	Wk 24	Wk 48	BL	Wk 4	Wk 12	Wk 24	Wk 48
PSI‐4										
DC	33.6 (7.6)	30.2 (8.1)	29.8 (8.1)	31.0 (6.6)	28.7 (10.1)	34.7 (10.8)	32.4 (10.2)	31.6 (9.8)	29.9 (8.7)	29.7 (8.9)
Total	89.0 (19.8)	77.4 (22.0)	77.9 (22.3)	79.5 (19.1)	78.2 (25.1)	90.8 (28.8)	86.5 (30.2)	85.1 (28.3)	78.4 (24.9)	80.1 (27.1)
PSOC	72.1 (11.1)	77.7 (11.0)	77.7 (12.1)	77.0 (11.1)	77.8 (12.8)	72.7 (12.3)	72.7 (11.2)	73.6 (12.4)	76.1 (13.4)	78.1 (15.0)

Abbreviations: BL, baseline; DC, difficult child subscale; PSI‐4 SF, parenting stress index 4th edition, short form; PSOC, parenting sense of competence; Wk, week.

**TABLE 3:** Differences within‐group for each timepoint.

	Change BL to Wk 4			Change BL to Wk 12			Change BL to Wk 24			Change BL to Wk 48		
	LSM diff	*p*	E. size	LSM diff	*p*	E. size	LSM diff	*p*	E. size	LSM diff	*p*	E. size
PSI‐4 SF												
DC												
MAYAC	−3.74	**	−0.91	−3.71	**	−0.67	−2.91	*	−0.61	−5.14	***	−0.88
CBI	−1.98	0.10	−0.46	−2.93	*	−0.44	−3.24	*	−0.40	−3.74	**	−0.47
Total												
MAYAC	−11.78	***	−0.94	−10.48	**	−0.68	−9.80	**	−0.78	−10.87	**	−0.57
CBI	−3.12	0.35	−0.33	−5.12	0.13	−0.31	−7.49	*	−0.45	−5.72	0.14	−0.30
PSOC												
MAYAC	5.45	**	0.75	5.76	***	0.79	5.56	**	0.74	5.58	**	0.61
CBI	0.24	0.89	0.08	2.37	0.17	0.28	2.97	0.09	0.28	4.47	*	0.51

Abbreviations: E. Size, effect size; LSM Diff, differences of least squares means; *p*, *p*‐value.

Note: p* < 0.05; p** < 0.01; p*** < 0.001.

### 3.4 Total Score

**FIGURE 3:**
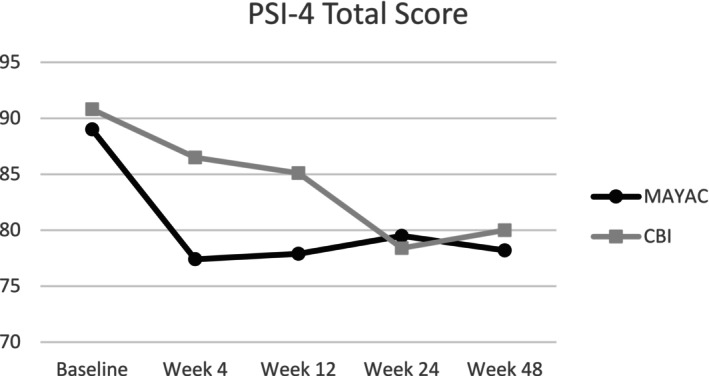
Changes in mean Psi‐4 total score.

As with the PSI‐DC scale, the Total Score for both MAYAC and CBI groups decreased from baseline through follow‐up, indicating a decrease in total stress (see Table 2). There were no significant differences between the MAYAC and CBI groups at any timepoint, nor was there an interaction effect between time and treatment. Within‐group differences (see Table 3) show that parents in both groups experienced less total stress from Baseline to Week 24. A significant difference was seen within the MAYAC group for total stress scores from Baseline to Week 4, Week 12, and Week 48, a within‐group difference that the CBI group did not see. Figure 3 shows changes in mean PSI‐4 Total Score throughout intervention and follow‐up.

### 3.5 PSOC

**FIGURE 4:**
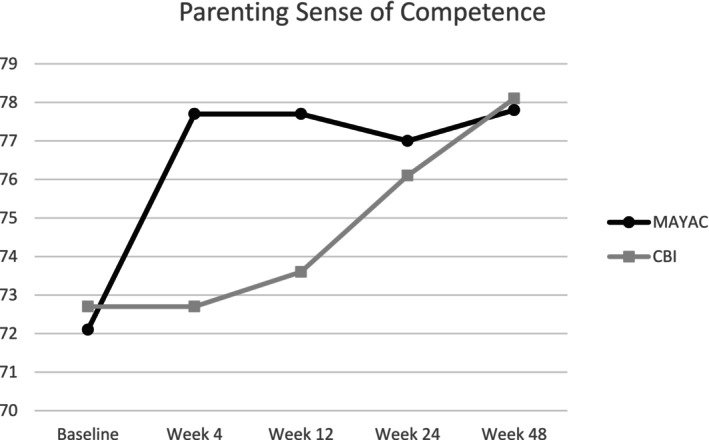
Changes in mean competence scores.

Raw scores on the PSOC were lowest at baseline for both MAYAC and CBI groups, indicating lower feelings of competence prior to starting treatment. Both groups saw an increase in competence by the end of the follow‐up (see Table 2). Parents in MAYAC saw significant within‐group differences in competence from Baseline to all assessment timepoints (Table 3), where competence increased. Parents in CBI only experienced a significant increase in competence from Baseline to follow‐up at Week 48. There were no significant between‐group differences on the PSOC (see Figure 4).

## 4 Discussion

We hypothesized that MAYAC would be superior in improving aspects of parent well‐being to include decreases in parental stress and increases in parental sense of competency. The rationale for this hypothesis was twofold. First, we hypothesized that an intervention requiring fewer hours and less intense treatment demands might be easier for families to incorporate into their daily lives. Second, we hypothesized that the structured parent training in MAYAC, which emphasized teaching skills in everyday family routines, would give parents more confidence in their ability to help their children, thereby reducing overall levels of stress.

Parent stress and competence outcomes were equivalent for both the CBI and MAYAC groups by the end of their intervention and at follow‐up assessments. While the type and focus of parent training differed between groups, one similarity is that the same intervention providers led parent training in both MAYAC and CBI. This trend in parent outcomes could also be due to participants in both groups receiving comparable time in parent training (approximately 30 min weekly). That said, parents of children in CBI received more time in parent training than may occur nationally for military families of autistic children; TRICARE reported a total average of 8 h of parent training over the course of a year (Department of Defense 2024), where participants in CBI received an average cumulative total of approximately 12 h across 6 months of their participation. The equivalent outcomes in both interventions highlight the value of parent involvement in addressing improvements in stress and competence. Taken together with results from our larger trial that showed that the two approaches were comparable in improving child adaptive behaviors and overall autism severity (Anderson et al. 2024), MAYAC might be a suitable alternative to traditional CBI for some families, especially considering the less intense treatment demands.

While there were no significant differences between groups on these parent measures, there were within‐group changes of note in both treatment arms over the course of the trial that could have implications for treatment decisions. The PSI‐DC effect size of −0.61 for MAYAC and −0.40 for CBI at the end of the intervention (Week 24) suggests that interventions targeting broad adaptive skills of varying intensity and parent involvement both lead to lower parental stress. This is similar to previous research findings suggesting that child participation in CBI decreases parental distress and raises parental ratings of self‐efficacy, irrespective of the amount of parent coaching received (D'Entremont et al. 2022). The significant decrease on the PSI‐DC for parents in MAYAC from Baseline to Week 4 is of note, as this pattern was not seen for parents in CBI. The PSI Total Score also saw significant decreases in parental stress within the MAYAC group, but not CBI, from Baseline to Week 4 or from Baseline to Week 12. By Week 24, the two groups were comparable in the overall decrease in parent stress on the PSI‐DC and PSI Total Score. While only speculative, it may have been that the structured parent‐mediated aspect of MAYAC, as opposed to the treatment‐as‐usual parent training for CBI, may have led to more rapid reductions in parent stress at the beginning of the trial. In addition, emphasis on teaching parents how to engage their child and encourage communication from the beginning of participation may have been beneficial. Future research should examine the effects of a coaching‐based intervention on family stress, as well as the relative benefits of providing parent‐mediated intervention focused on engagement and communication initially (e.g., Ingersoll and Wainer 2013).

Like the findings on parent stress, there were significant within‐group differences for both MAYAC and CBI on the PSOC, but no between‐group differences. A more rapid change in the first weeks of the trial for the MAYAC group was revealed; in this case an increase in their sense of competence. This significant within‐group difference was also true for the MAYAC group from Baseline to Week 12 and Baseline to Week 24, but changes in CBI competence during these times were not significantly different from Baseline. The two groups were similar at follow‐up, where both interventions resulted in parents feeling significantly more confident in their parenting role than at Baseline. The parent‐mediated focus of MAYAC might have given parents the tools earlier on to feel more competent at a faster rate, while it took more time for the competence to grow within CBI. D'Entremont et al. compared two methods to providing CBI and found that when dedicated parent coaching was delivered, parents reported significantly higher ratings of parent satisfaction when it came to a feeling of “learning helpful strategies” (2022). Learning the strategies provided in parent coaching within MAYAC may have led to an earlier increased sense of competence.

While parent training time was equivalent for both groups throughout the intervention, it is possible that the ratio of parent‐mediated intervention to total hours delivered influenced the more rapid shift in stress and competence for MAYAC from Baseline to the first assessment timepoint. Parent training accounted for approximately 12% of total intervention hours in the MAYAC group, and around 4% of total intervention hours for the CBI group. More dedicated time receiving structured parent training in the MAYAC group could explain the significant within‐group decrease in stress and increase in competence earlier in the trial.

Collectively, the findings from this relatively small trial suggest there is a benefit to parents when their young autistic child receives quality behavioral analytic interventions and parents themselves also participate. This is consistent with other findings of parent outcomes from children's participation in CBIs of varying models and intensity (D'Entremont et al. 2022; Kasari et al. 2015). While this study did not have a stand‐alone parent‐mediated intervention, it does lend credence to the value of parental involvement. While beyond the scope of this RCT, it may be that the systematic participation of parents with a lower “dose” of intervention may be preferred by some parents and may be as effective for some autistic children. The added value of parent‐mediated interventions has gained considerable traction as an effective model in recent years (Ingersoll et al. 2020; Kasari et al. 2015).

While this trial demonstrates positive parent outcomes of young autistic children receiving one of two approaches to behavioral analytic intervention, there are admittedly limitations. Most notably, the COVID‐19 pandemic adversely impacted recruitment for this trial and the frequency and methods of in‐person interventions as it did many RCTs during this time frame (Audisio et al. 2022). Clinics and research teams were mandated to pause within months of initial recruitment and enrollment of the trial. When clinics were allowed to reopen, many pandemic restrictions remained that created barriers for the RCT, such as limited in‐person contact, mandatory masking and distancing protocols, and strict health monitoring resulting in frequent cancellations. Families, too, exercised caution during the time of uncertainty as they wished to minimize exposure to illness. This resulted in lower intervention hours than anticipated (although still comparable to actual intervention hours delivered in community settings). Additionally, the parent outcome measures were self‐report and limited in number to minimize participant burden in the context of a trial with many child outcome measures as well. No objective measures such as physiological markers of parent stress were included. Furthermore, all families in this trial had at least one caregiver in the military, and most families were white, well‐educated, and not receiving additional assistance (i.e., food stamps, WIC, etc.). Future work should include a larger trial with more diverse participants outside of the military population. In addition, it would be beneficial for future trials to extend intervention and follow‐up periods.

In conclusion, this RCT comparing two behavioral analytic approaches in a sample of young military dependents demonstrated significant parent stress at the beginning of the trial. In the context of a trial that hypothesized that a parent‐involved, lower‐dosed intervention (MAYAC) would not be inferior to a comprehensive behavioral intervention (CBI), it is not surprising that both interventions had a positive impact on parents. However, it is also noteworthy that self‐reported parental stress decreased more rapidly, and parental sense of competency increased at a steeper rate when there was a greater focus on structured parent training, as was delivered in MAYAC. These trends in parent stress reduction and increased parenting sense of competence represent an important observation and could suggest value in incorporating parent training regardless of an overall approach to behavioral intervention.

## Conflicts of Interest

Some of the published materials to be used in the interventions have been developed by the investigators, for which they may receive royalties. This was disclosed to participants through informed consent.

## Data Availability

The data that support the findings of this study are openly available in NDA at https://nda.nih.gov/.
